# Safety and efficacy of oral edible bird’s nest supplementation: Anti-inflammatory and immunomodulatory benefits for Arabian race stallions during exercise

**DOI:** 10.14202/vetworld.2025.178-188

**Published:** 2025-01-27

**Authors:** Khalid Obaid AL-Khaldi, Khalid Hamed Al-Ruzaiqi, Abdul Salam Babji, Seng Joe Lim, Mohammed Babatunde Sadiq, Nurhusien Yimer

**Affiliations:** 1 Veterinary Services Administration, Mounted Police Headquarter, Muscat, Sultanate of Oman; 2 Department of Food Sciences, Faculty of Science and Technology, Universiti Kebangsaan Malaysia, 43600 UKM Bangi, Selangor, Malaysia; 3 Innovation Centre for Confectionery Technology (MANIS), Faculty of Science and Technology, Universiti Kebangsaan Malaysia, 43600 UKM Bangi, Selangor, Malaysia; 4 Department of Farm and Exotic Animal Medicine and Surgery, Faculty of Veterinary Medicine, Universiti Putra Malaysia, 43300, Serdang, Selangor, Malaysia; 5 Department of Veterinary Sciences, School of Medicine, IMU University, Bukit Jalil, 57000 Kuala Lumpur, Malaysia; 6 Veterinary Reproduction Division, Faculty of Veterinary Medicine, Airlangga University, Surabaya 60115, Indonesia

**Keywords:** anti-inflammatory, arabian race stallions, edible bird’s nest, exercise stress, immunomodulatory

## Abstract

**Background and Aim:**

Exercise-induced oxidative stress and inflammation adversely affect the health and performance of athletic horses. This study aimed to evaluate the safety of edible bird’s nest (EBN) supplementation and its potential anti-inflammatory and immunomodulatory effects in Arabian race stallions during exercise. Key objectives included assessing EBN’s impact on hepatic and renal function, hematological profiles, and sialic acid (SA) levels.

**Materials and Methods:**

Two experiments were conducted on 18 Arabian stallions. In Experiment 1, six healthy stallions were divided into control (n = 2) and EBN-supplemented groups (n = 4) to assess safety. The treatment group received 10 g of EBN daily for 12 days. Vital signs, hematological parameters, and organ function markers were monitored. In Experiment 2, 12 stallions were divided into three groups: EBN (n = 3), Premier E^®^ (n = 3), and control (n = 6). The exercise regimen included a daily 10-min walk, 10-min canter (30 km/h), and 10-min walk for 30 days. Blood samples were analyzed for hematological indices and SA levels pre- and post-exercise.

**Results:**

In Experiment 1, EBN supplementation demonstrated no adverse effects, maintaining normal hematological and vital parameters. Kidney and liver function tests revealed significantly reduced creatine kinase, total bilirubin, and aspartate aminotransferase levels in the EBN group. Experiment 2 showed higher SA levels post-exercise in the EBN group (p < 0.05) compared to Premier E^®^ and control groups, suggesting enhanced anti-inflammatory response. Hematological indices, including mean corpuscular volume, red cell distribution width, and platelet counts, were significantly improved in the EBN group, indicating potential immunomodulatory benefits.

**Conclusion:**

EBN supplementation is safe and offers anti-inflammatory and immunomodulatory effects in Arabian race stallions, reducing exercise-induced hepatic and muscular stress markers while enhancing recovery. These findings support EBN as a valuable dietary supplement for performance horses. Further studies should explore long-term effects and molecular mechanisms to optimize its use in equine sports.

## INTRODUCTION

Purebred Arabian racing stallions are of great economic importance to the spread of Arabian racing horses worldwide. Arab racing horses are distinguished by their distinct breeds, which are inherited from fathers and grandfathers, and descend from blood-bearing genetic characteristics that make them withstand high speeds and high effort [1]. Among the founding Arabian horses of racing horses was the horse “Zad AL-Rakib,” which was given as a gift to the Al Azd people of Oman for more than 3000 years [2, 3]. With their incredible stamina, Arabian horses exhibit several anatomical and functional adaptations for athletic performance [4]. Arabian horses can maintain good health, rarely get ill, consume less than other breeds, and have great endurance for lengthy treks [5, 6].

In horses, exercise has varying effects on their physiological and metabolic functions [7]. Depending on the type, intensity, duration, environmental conditions, health status, and training status, exercised horses may have an oxidant/antioxidant imbalance [8]. Hematological and biochemical basal values are also altered in exercising horses under various climatic and working conditions [8].

Nutritional supplements are increasingly being produced and used in animals for their nutritional benefits, improved physiological function, and health enhancement [9, 10]. For instance, Li *et al*. [7] found that supplementing Yili horses’ diet with 50 g of L-citrulline increased the plasma concentrations of arginine and citrulline, which led to improved athletic performance. Edible bird’s nest [EBN], a product from the salivary secretions of swiftlet species, is a well-known valued product among the Chinese and has been consumed for centuries due to its medicinal and nutritional properties, including anti-aging, anti-oxidative, anti-cancer, and anti-inflammatory [11, 12]. EBN is rich in sialic acid (SA), which is a biological constituent with metabolic enhancing and antioxidative properties and has positive effects on equine health [12]. While nutritional supplements are commonly used to enhance the health and performance of racehorses, there is limited research on the safety and efficacy of (EBN) supplementation in Arabian race stallions, particularly in reducing exercise-induced inflammation and supporting immune function.

Since oxidative and associated immune system changes are well-recognized in exercised horses, EBN can ameliorate such oxidative changes, thereby restoring homeostasis in horses exposed to physical activity. Given the international scope of the equine industry, with the continuous trade of horses and the increasing exchange of genetic material in racehorse breeding [13], poor performance or failure to compete at all due to exercise-induced physiological fatigue could affect the animal’s economic value, as well as the value of its ova/semen. Therefore, understanding the potential impacts of EBN supplementation on equine athletes could provide new strategies for managing the health and improving the performance ability of racehorses, which is of great interest to both riders and horse breeders in the equine industry.

This study aimed to evaluate the safety of oral EBN supplementation and assess its potential anti-inflammatory and immunomodulatory effects on liver and kidney function and hematological profiles in Arabian race stallions undergoing regular exercise.

## MATERIALS AND METHODS

### Ethical approval

The study was approved by the Animal Welfare Committee at the Animal Health Research Center (Approval number: 15), following the guidelines for the humane treatment of animals outlined by the Ministry of Agriculture and Fisheries in Oman.

### Study period and location

This study was conducted from April to May 2019 in Muscat, the Sultanate of Oman.

### Animals

Eighteen stallions, 6–16 years old, regularly participating in racing events and certified healthy by a veterinarian, were conveniently selected for this study. The horses are routinely exposed to fitness training throughout the year and are allowed to rest 1 day per week. This training started at 6 am daily and lasted until 10 am. This daily training program was similar for all the horses, which entailed a warm-up (10 min walk, then 10 min canter (30 km/h) then 10 min walk). The horses did not participate in any official competitions during the study period, which was conducted between April and May 2019.

### Housing and diet

The horses were housed in the same environment: 20 m^2^ area stables at the Mounted Police Division in the Royal Oman Police area, Muscat, Oman. All were provided with food and water *ad libitum*. The daily diet consisted of 8 kg of Katambora dray grass, 1 kg of oats, and 2 kg of concentrated ration cubes of HAVENS^®^ (Netherlands) basis-sport condition cubes (carbohydrates 51%, crude protein 12.6%, crude fat 3.4%, calcium [Ca] 10.4 g, and phosphorus 4.7). The oats and concentrated ration cubes were given in two separate feedings with the same quantity in each meal, at 8 h interval between feedings.

### Experiment 1: Safety assessment of EBN

Six normal healthy stallions were divided into two groups (control; n = 2, treatment group = 4). The treatment group received 10 g of EBN orally through a syringe once a day (in the morning before meal and exercise) for 12 days. Before oral supplementation, all horses were examined for rectal temperature, respiratory rate, and pulse rate during the same period. Blood sampling was conducted for hematology at weeks 0, 1, and 2 of the study periods. On completing the 12-day trial, the same veterinarian performed the final physical examination. The vital signs of the horses were observed daily and blood samples were collected weekly.

Blood samples (5 mL) were collected from the jugular vein using a vacutainer (Vacutainer^®^, Becton Dickenson, USA). Two samples were collected before and after the exercise. Both samples were coagulated for 30 min and centrifuged at 822× *g* for 15 min. The samples were stored in cooling bags at 4°C, transported to the laboratory, and stored at –80°C until further analysis. Subsequent sampling was performed at weeks 1, 2, and 3 after supplementation.

The SmartLyte electrolyte analyzer (Diamond Diagnostics, Germany) was used to measure the concentrations of sodium, potassium, and other electrolytes. Meanwhile, the concentrations of urea, creatinine, glucose, triglycerides, cholesterol, Ca, and magnesium and activities of aspartate aminotransferase (AST), creatine kinase (CK), and lactate dehydrogenase in blood serum were analyzed with (Beckman Coulter, Inc. United States) commercial kits using the Olympus AU640 (Olympus, Japan) biochemical analyzer. We obtained reference values from the equine reference intervals used by the Veterinary Department, Royal Oman Police, Mounted Police Division, Muscat, Sultanate of Oman.

### Experiment 2: Effect oral supplementation with EBN and Premier E^®^ (Equine products UK Ltd.)

In the first treatment group, three horses were selected (n = 3) and given 21 g of spray-dried EBN (EBNSD) dissolved in 100 mL of distilled water daily for 30 days. A dosage of 21 g of EBN was selected, guided by its reported antioxidant properties, similar to Premier E®, demonstrating its effectiveness in horses. For instance, Burd *et al*. [14] selected a 12-day supplementation period to assess immediate (acute) and short-term effects on muscle protein synthesis. The EBN composition is summarized in Supplementary Table 1. In the second treatment group, three horses were selected (n = 3) and given 25 g of Premier E^®^ (Vitamin E α–tocopheryl acetate 30,000 IU, lysine 27500 mg, methionine 10,000 mg, and selenium 10 mg) dissolved in distilled water daily for 30 days (n =3). The composition of Premier E^®^ used in this experiment is presented in Supplementary Table 2. The third group was the control (n = 6). The treatment was conducted every morning at 6:00 am before morning exercise.

**Supplementary Table 1:** Comparison of Composition bioactive edible bird’s nests (EBN) raw clean (control) and clean hydrolysate edible birds nest’s spray-dried (EBNSD) and analyzed in UKM UNIPEQ MALAYSIA.

Test description	Raw clean EBN	Hydrolysate Spray dried EBNSD	Test method
Total Ash (g/100 g)	2.14	4.38	In-house method No: STP/Chem/A05 based on AOAC 20^th^ Edition: 923.03
Moisture (g/100 g)	17.48	15.35	In-house method No: STP/Chem/A04 based on AOAC 20^th^ Edition: 950.46
Protein (g/100 g)	56.88	57.16	In-house method No: STP/Chem/A03 based on AOAC 20^th^ Edition: 981.10
Total Fat-Soxhlet (g/100 g)	0.20	0.20	In-house method No: STP/Chem/A02 based on AOAC 20^th^ Edition: 991.36
Carbohydrate (g/100 g)	23.3	22.91	In-house method No: STP/Chem/A06 based on Promerance Food Analysis: Therory and Practice, 2^nd^ Ed. (pg 637)
Energy (by calculation) (kcal/100 g)	323	322	In-house method No: STP/Chem/A01 based on Pearson’s The Chemical Analysis of Foods (6^th^ Edition, page 578)
Sialic acid %	10	10.2	HPLC

EBN=Edible bird’s nest

**Supplementary Table 2:** Composition of premier E as a supplement for horses (Equine products UK Ltd.)

Constitutent	Quantity
Vitamin E (as α-tocopheryl acetate)	60,000 IU
Lysine	55,000 mg
Methionine	20,000 mg
Trace elements	
E8 Selenium (as sodium selenite)	20 mg
Analytical constituents	
Crude protein	7.5%
Crude ash	5%
Crude oil	6.42%
Crude fiber	<0.1%
Sodium	0.03%

### Exercise test

The study design consisted of one exercise test per training program. The exercise test was performed on a track with a sandy surface. Exercise tests and general animal care were performed by professional staff who were not part of the research team. The exercise was scheduled for a warm-up (10 min walk, 10 min canter, 10 min walk) daily except on Fridays of the 30 days trial. The canter was to slow run (three-beat gait/slow gallop) for 10 min (30 km/h). Previously, the stallions were trained to run together during cantering. However, the horses were made to run/walk differently during the experiment. On the other days, the horses were left to walk around in the stables without any vigorous activity.

### Blood sampling and SA assessment

Blood samples were collected from the jugular veins of the horses before and 30 min after the weekly exercise test. An HM5 analyzer (Abaxis Europe - German) was used for hematological profiling.

The enzyme-linked immunosorbent assay (ELISA) was used to measure SA concentrations with commercially available ELISA kits (Bioassay Technology Laboratory, Shanghai, China). Blood samples were collected in tubes containing potassium K-ethylenediaminetetraacetate (K-EDTA) and stored on ice at 4°C for preservation.

Centrifugation was conducted at 3,000× *g* for 15 min. Thereafter, plasma was extracted, and five volumes of saline solution were used to wash the erythrocyte suspension (1 volume) 3 times before being centrifuged at 3,000× *g* for 15 min. All plasma aliquots were frozen and stored at –26°C until ELISA was performed. The SA concentration was measured using a commercially available ELISA kit (Beckman Colter Inc.). The reaction was terminated by the addition of an acidic stop solution, and absorbance was measured at 450 nm.

### Hematological assays

Routine hematological parameters were analyzed after 4 weeks for all horses in each group. An automated hematology analyzer was used to measure and count the following parameters: Red blood cells (RBC), hematocrit (HCT), hemoglobin (HGB) concentration, mean corpuscular hemoglobin concentration (MCHC), mean corpuscular volume (MCV), mean corpuscular hemoglobin (MCH), red cell distribution width (RDW), and platelet count (PLT). The same analyzer was used to measure the white blood cell counts (neutrophils, lymphocytes, monocytes, basophils, and eosinophils).

### Statistical analysis

The statistical analysis was conducted using IBM SPSS Software Version 23.0 (IBM Corp., NY, USA). Data normality was verified using the Shapiro–Wilk test. Results are expressed as mean ± standard deviation (SD), and differences were considered significant at p < 0.05. Repeated measures analysis of variance (ANOVA) was applied to evaluate longitudinal data across different time points, comparing vital parameters, hematological indices, and biochemical markers between treatment and control groups. One-way ANOVA with Tukey’s *post hoc* test was used for between-group comparisons of hematological indices and post-exercise sialic acid levels. Electrolyte and metabolic marker stability were assessed using ANOVA to confirm no significant variations across groups. The statistical methods ensured robust comparisons and identification of significant changes related to EBN supplementation.

## RESULTS

### Safety of oral EBN supplementation

In the first experiment, oral supplementation of horses with EBN did not affect any of the vital parameters of the 12-day trial. There were no significant differences in the pulse rate, rectal temperature, and respiratory rate between treatments (Table 1). Likewise, hematological parameters were maintained at weeks 0, 1, and 2 of the study periods. All hematological parameters were within normal limits for healthy horses throughout the experimental period (Table 2).

**Table 1:** Vital parameters in the EBN and control groups at the end of the 12-day trial.

Parameters	Baseline (+SD)	Day 12
Rectal temperature (°C)		
EBN	36.9 ± 0.1^A^	37.0 ± 0.1^A^
Control	37.1 ± 0.2^A^	36.9 ± 0.1^A^
Pulse rate (beats/min)		
EBN	33.0 ± 2.0^A^	30.5 ± 1.0^A^
Control	33.0 ± 1.4^A^	33.0 ± 1.4^A^
Respiratory rate (cycles/min)		
EBN	20.5 ± 4.1^A^	18.5 ± 1.0^A^
Control	19.0 ± 0.7^A^	20.0 ± 1.0^A^

Values are presented in mean ± standard deviation, and those with the same superscripts do not significantly differ (p < 0.05). EBN=Edible bird’s nest

**Table 2:** Comparison of hematological indices between horses supplemented with oral EBN and control groups at baseline (week 0), week 1, and week 2.

Indices	Group	Week 0 (before supplementation)	Week 1	Week 2	Normal range
WBC (*10^9^/L)	EBN	5.00 ± 0.96^A^	5.84 ± 0.68^A^	6.08 ± 0.31^A^	5.14–9.52
	Control	3.64 ± 0.71^A^	3.46 ± 0.69^A^	4.0 ± 0.21^A^	
Lymphocytes (10^3^/μL)	EBN	1.48 ± 0.27^A^	1.85 ± 0.34^A^	1.77 ± 0.19^A^	4.9–39.9
	Control	1.03 ± 0.09^A^	1.12 ± 0.26^A^	1.35 ± 0.18^A^	
Monocytes (10^3^/μL)	EBN	0.18 ± 0.03^A^	0.22 ± 0.06^A^	0.15 ± 0.05^A^	
	Control	0.18 ± 0.06^A^	0.13 ± 0.04^A^	0.17 ± 0.03^A^	
Neutrophils (10^3^/μL)	EBN	3.24 ± 0.30^A^	3.68 ± 0.39^A^	4.04 ± 0.29^A^	2.6–6.4
	Control	2.37 ± 0.23^A^	2.16 ± 0.43^A^	2.41 ± 0.50^A^	
Eosinophils (10^3^/μL)	EBN	0.09 ± 0.01^A^	0.08 ± 0.00^A^	0.10 ± 0.01^A^	0–0
	Control	0.03 ± 0.00^A^	0.03 ± 0.00^A^	0.04 ± 0.00^A^	
Basophils (10^3^/μL)	EBN	0.01 ± 0.00^A^	0.01 ± 0.00^A^	0.02 ± 0.00^A^	0–0
	Control	0.01 ± 0.00^A^	0.00 ± 0.00^A^	0.00 ± 0.00^A^	
RBC	EBN	10.28 ± 0.68^A^	9.38 ± 0.50^A^	9.08 ± 0.51^A^	5.6–8.6
	Control	11.6 ± 1.69^A^	10.12 ± 1.20^A^	9.94 ± 1.40^A^	
HGB (g/dL)	EBN	18.70 ± 1.55^A^	16.3 ± 0.91^A^	14.87 ± 0.85^A^	10.2–19.5
	Control	21.60 ± 6.76^A^	18.1 ± 2.67^A^	17.2 ± 2.61^A^	
HCT	EBN	51.92 ± 3.51^A^	47.51 ± 2.00^A^	46.25 ± 1.64^A^	28.0–43.0
	Control	59.66 ± 1.52^A^	51.86 ± 5.80^A^	49.97 ± 5.40^A^	
MCV (fL)	EBN	50.5 ± 0.68^A^	50.75 ± 0.94^A^	51.25 ± 1.10^A^	45–55
	Control	49.96 ± 1.53^A^	51.33 ± 1.04^A^	50.5 ± 1.76^A^	
MCH (pg)	EBN	18.11 ± 0.51^A^	17.35 ± 0.36^A^	16.32 ± 0.18^A^	16–20
	Control	15.37 ± 3.43^A^	17.73 ± 0.76^A^	17.2 ± 0.21^A^	
MCHC (g/dL)	EBN	35.88 ± 0.83^A^	34.22 ± 0.53^A^	32.05 ± 0.69^A^	35–37
	Control	30.60 ± 6.89^A^	34.5 ± 1.37^A^	34.05 ± 1.52^A^	
RDW (%)	EBN	22.95 ± 0.36^A^	22.15 ± 0.47^A^	21.95 ± 0.25^A^	
	Control	19.40 ± 4.45^A^	22.0 ± 0.90^A^	21.85 ± 1.16^A^	
PLT (uL)	EBN	117.83 ± 17.13^A^	138.75 ± 13.59B	147.25 ± 13.9B	100–210
	Control	147.60 ± 66.96^A^	99.33 ± 9.30B	109.5 ± 18.73B	

Values that are significantly different (p < 0.05) from baseline (week 0) are presented with different superscript letters. Values with the same superscript letter were not significantly different from baseline (week 0). All values are presented in mean ± standard deviation. RBC=Red blood cell count, HBG=Hemoglobin, HCT=Hematocrit, MCV=Mean corpuscular volume, MCH=Mean corpuscular hemoglobin, MCHC=Mean corpuscular hemoglobin concentration, RDW=Red cell distribution width, PLT=Platelets count, EBN=Edible bird’s nest

Overall, neither the electrolyte indices nor liver and kidney test parameters significantly differed between the groups before and 1 week after the intervention. As shown in Table 3, EBN supplementation did not significantly affect chloride, CO_2_, potassium, or Ca levels, suggesting a stable electrolyte balance compared with the control.

**Table 3:** Comparison of electrolyte concentrations between the EBN and control groups before and after supplementation.

Parameters	Reference	Before supplementation		Week 1		Week 2		Week 3	
			
		Control	EBN	Control	EBN	Control	EBN	Control	EBN
				
Group		A (EBN)	B (Control)	A (EBN)	B (Control)	A	B	A	B
Sodium (mmol/L)	134–142	134.50 ± 0.29^A^	134.75 ± 0.85^A^	131.50 ± 2.02^A^	135.00 ± 1.73^A^	132.00 ± 0.58^A^	135.00 ± 0.00^a^	136.00 ± 0.00^a^	96.50 ± 32.40^b^
Potassium (mmol/L)	3.0–5.0	3.65 ± 0.14^a^	3.70 ± 0.11^a^	3.60 ± 0.00^a^	3.78 ± 0.05^a^	3.45 ± 0.03^a^	3.08 ± 0.51^a^	3.80 ± 0.12^a^	3.35 ± 0.56^a^
Chloride (mmol/L)	95–103	100.00 ± 0.00^a^	97.5 ± 0.96^a^	95.00 ± 1.15^a^	96.75 ± 1.31^a^	95.50 ± 0.87^a^	79.75 ± 10.28^a^	97.50 ± 0.29^a^	82.00 ± 10.06^a^
CO_2_ (mmol/L)		23.50 ± 0.29^a^	26.75 ± 0.75^a^	25.50 ± 0.87^a^	27.50 ± 1.04^a^	26.00 ± 0.00^a^	22.50 ± 3.18^a^	25.00 ± 0.58^a^	25.25 ± 4.94^a^
Calcium (mgl/dL)	9.0–14.0	12.25 ± 0.14^a^	11.98 ± 0.23^a^	11.35 ± 0.26^a^	11.95 ± 0.03^a^	11.45 ± 0.09^a^	10.00 ± 1.24^a^	11.95 ± 0.26^a^	10.03 ± 1.35^a^
Iron (ng/mL)	20–450	133.50 ± 3.18^a^	156.75 ± 18.25^a^	148.50 ± 12.99^a^	169.00 ± 21.40^a^	**115.50 ± 8.1 5^a^**	**149.00 ± 1.73^b^**	**140.75 ± 25.87^A^**	**220.00 ± 40.99^B^**
Phosphate (mmol/L)	0.9–1.9	2.90 ± 0.00^a^	3.60 ± 0.34^a^	2.85 ± 0.38^a^	3.38 ± 0.15^a^	3.40 ± 0.12^a^	2.78 ± 0.21^a^	3.65 ± 0.03^a^	3.08 ± 0.47^a^

Values presented in bold are statistically different between the groups. EBN=Edible bird’s nest

Regarding kidney test parameters, CK levels were significantly lower in the EBN-supplemented group compared with the control group at weeks 2 and 3 (Table 4). In the liver test, the EBN group had significantly lower total bilirubin and AST levels at weeks 2 and 3 post-intervention compared with the control (Table 5).

**Table 4:** Comparison of kidney function parameters between the EBN and control groups before and after supplementation.

Parameters	Reference	Before supplementation		Week 1		Week 2		Week 3	
			
		Control	EBN	Control	EBN	Control	EBN	Control	EBN
Urea (mmol/L)	2.5–10.0	15.50 ± 0.87^A^	15.00 ± 1.29^A^	14.00 ± 0.58^a^	14.25 ± 1.49^a^	15.00 ± 0.58^a^	11.50 ± 2.10^a^	13.50 ± 0.29^a^	12.50 ± 1.85^a^
Creatinine (μmol/L)	0.85–1.65	1.17 ± 0.03^A^	1.31 ± 0.03^a^	1.13 ± 0.04^a^	1.34 ± 0.05^a^	**1.25 ± 0.09^a^**	**1.09 ± 0.16^a^**	**1.19 ± 0.01^a^**	**1.13 ± 0.17^a^**
CK (U/L)	110–250	159.50 ± 8.37^a^	113.75 ± 8.46^a^	120.00 ± 3.46^a^	112.75 ± 10.44^a^	134.50 ± 2.60^a^	92.50 ± 11.43^b^	155.00 ± 6.93^A^	96.25 ± 13.51^B^

Values presented in bold are statistically different between the groups. EBN=Edible bird’s nest

**Table 5:** Comparison of liver function parameters between the EBN and control groups before and after supplementation.

Parameters	Reference	Week 0		Week 1		Week 2		Week 3	

		Control	EBN	Control	EBN	Control	EBN	Control	EBN
TP	5.3–7.3	6.50 ± 0.06^a^	6.33 ± 0.09^a^	5.75 ± 0.14^a^	6.28 ± 0.09^a^	6.20 ± 0.00^a^	5.28 ± 0.65^a^	6.35 ± 0.09^a^	5.43 ± 0.77^a^
Albumin	2.9–4.1	2.64 ± 0.05^a^	2.67 ± 0.03^a^	2.33 ± 0.01^a^	2.69 ± 0.07^a^	2.56 ± 0.06^a^	2.22 ± 0.24^a^	2.64 ± 0.01^a^	2.31 ± 0.30^a^
Total bilirubin	1.3–3.4	2.75 ± 0.14^a^	2.23 ± 0.09^a^	2.30 ± 0.06^a^	2.15 ± 0.18^a^	**2.60 ± 0.17^a^**	**1.78 ± 0.26^b^**	**2.85 ± 0.09^a^**	**1.90 ± 0.25^b^**
AST (U/L)	168–494	233.00 ± 10.97^a^	208.00 ± 27.94^a^	196.00 ± 12.70^a^	199.75 ± 20.36^a^	**214.50 ± 17.61^a^**	**160.50 ± 22.90^b^**	**219.00 ± 21.36^a^**	**164.00 ± 28.30^b^**
ALT (U/L)	0–6	9.50 ± 0.87^a^	9.00 ± 1.35^a^	7.50 ± 0.87^a^	7.75 ± 0.48^a^	8.50 ± 0.87^a^	6.25 ± 0.85^a^	10.50 ± 1.44^a^	7.00 ± 0.71^a^
ALP (U/L)	86–285	66.50 ± 1.44^a^	68.75 ± 4.13^a^	58.00 ± 4.62^a^	70.00 ± 4.04^a^	66.00 ± 6.35^a^	55.25 ± 3.90^a^	64.00 ± 5.20^a^	57.50 ± 8.63^a^
GGT	1.0–40.0	20.00 ± 3.46^a^	14.75 ± 1.93^a^	16.00 ± 3.46^a^	14.50 ± 1.85^a^	31.00 ± 11.55^a^	12.25 ± 1.31^b^	27.50 ± 10.10^a^	12.25 ± 1.93^b^
LDH	100–350	241.00 ± 7.51^a^	170.75 ± 37.75^b^	188.50 ± 10.10^a^	167.00 ± 36.03^a^	185.00 ± 1.15^a^	128.75 ± 13.97^b^	201.50 ± 2.60^a^	148.75 ± 44.46^b^
Triglycerides (mg/dL)	2–41	27.50 ± 3.75^a^	15.50 ± 1.89^a^	15.50 ± 2.60^a^	19.00 ± 0.71^a^	17.50 ± 3.75^a^	16.25 ± 2.81^a^	22.00 ± 4.62^a^	19.00 ± 3.67^a^
Cholesterol (mg/dL)	51–129	81.00 ± 5.77^a^	79.75 ± 2.93^a^	69.50 ± 2.60^a^	83.25 ± 2.06^a^	80.50 ± 6.06^a^	69.75 ± 9.44^a^	76.50 ± 0.87^a^	76.25 ± 11.67^a^
Uric acid		0.59 ± 0.01^a^	0.35 ± 0.20^a^	0.00 ± 0.00^a^	0.13 ± 0.13^a^	0.27 ± 0.16^a^	0.00 ± 0.00^a^	0.67 ± 0.04	0.27 ± 0.15^a^
Glucose	4.3–5.5	92.05 ± 7.01^a^	91.35 ± 4.09^a^	84.25 ± 1.47^a^	92.10 ± 1.88^a^	86.00 ± 0.92^a^	74.53 ± 8.74^a^	92.60 ± 4.68^a^	75.75 ± 8.53^a^

Values presented in bold are statistically different between the groups. LDH=Lactate dehydrogenase

### Oral EBN supplementation and premier E^®^

In the second experiment, SA levels were not significantly different (p > 0.05) at each week (Weeks 1, 2, 3, and 4) between the three groups (Table 6) before exercise. This finding reflects the similarity between the groups and suitability for investigating the effects of EBN and premier E^®^ supplementation.

**Table 6:** Comparison of mean SA concentration (mg/dL) between horses supplemented with EBN, PremierE^®^, and control before and after exercise.

Week	Before exercise			After exercise		
	
	EBN	Premier E^®^	Control	EBN	Premier E^®^	Control
1	2.14 ± 0.64^A^,^A^	2.21 ± 0.38^A^,^A^	2.36 ± 0.01^A^,^A^	2.31 ± 0.72^A^,^A^	2.34 ± 0.55^A^,^A^	2.07 ± 0.22^A^,^A^
2	2.34 ± 0.27^A^,^A^	2.31 ± 0.25^A^,^A^	2.34 ± 0.02^A^,^A^	2.43 ± 0.32^A^,^A^	2.35 ± 0.26^A^,^A^	2.10 ± 0.29^A^,^A^
3	2.86 ± 0.47^A^,^A^	2.34 ± 0.36^A^,^A^	2.29 ± 0.05^A^,^A^	3.16 ± 0.75^A^, B	2.34 ± 0.25b,^A^	2.12 ± 0.21b,^A^

Means for each group along the columns are represented with upper case superscripts, whereas means across the rows are compared using lower case superscripts. Means with different superscripts differ significantly

Although both the EBN, premier E, and control groups exhibited similar SA levels pre-and post-exercise at weeks 1 and 2, the EBN-supplemented horses showed a notable increase in SA levels post-exercise at week 4 (p = 0.03), indicating an enhanced anti-inflammatory response.

Table 7 presents the hematological indices in the treatment and control groups after 4 weeks, with reference values obtained from Winnicka [15]. The PLTs and RDW in the control group were 10% and 5% higher than those in the EBN group (p < 0.05), indicating the potential thrombocyte stability benefits of EBN supplementation. Although the MCV was not significantly different between the horses supplemented with EBN and Premier E^®^, the values were significantly higher (p = 0.02) than in the control.

**Table 7:** Values of hematological indices of Arabian race stellions between groups supplemented with EBN, Premier E^®^, and control after exercise.

Indices	EBN	Premier E^®^	Control	Reference
RBC (*10^9^/L)	8.19 ± 0.78^a^	8.94 ± 0.62^a^	9.29 ± 1.65^a^	5.5–10.0
HGB (g/dL)	15.30 ± 1.95^a^	16.66 ± 1.13^a^	16.1 ± ± 3.42^a^	8–18
HCT	38.78 ± 4.26^a^	44.46 ± 3.50^a^	41.94 ± 4.84^a^	24–52
MCV (fL)	48.14 ± 1.84^a^	49.74 ± 2.24^a^	46.33 ± 1.75^b^	35–58
MCH (pg)	17.66 ± 1.08^a^	18.58 ± 0.59^a^	17.26 ± 0.83^a^	10–20
MCHC (g/dL)	37.24 ± 1.76^a^	37.52 ± 2.38^a^	37.26 ± 3.16^a^	31–37
RDW (%)	23.28 ± 1.39^a^	24.18 ± 1.94^a^	27.06 ± 3.00^b^	11–17
PLT (μL)	79.84 ± 36.90^a^	**115.52 ± 17.67^a^**	**261.66 ± 19.72^b^**	100–350
Neutrophils (10^3^/μL)	5.90 ± 3.03^a^	4.20 ± 1.12^b^	3.83 ± 0.55^b^	2.7–6.6
Lymphocytes (10^3^/μL)	1.78 ± 0.84^a^	1.13 ± 0.94^a^	1.61 ± 0.21^a^	1.2–4.9
Monocytes (10^3^/μL)	0.45 ± 0.32^a^	0.23 ± 0.13^a^	0.28 ± 0.13^a^	0–0.6
Eosinophils (10^3^/μL)	0.36 ± 0.16^a^	0.29 ± 0.26^a^	0.14 ± 0.04^a^	0–1.2
Basophils (10^3^/μL)	0.27 ± 0.36^a^	0.09 ± 0.08^a^	0.08 ± 0.12^a^	0–0.2

Reference values [15]. Values are presented in mean ± SEM, Values with different superscripts are statistically different. Values presented in bold are statistically different between the groups. RBC=Red blood cell count, HBG=Hemoglobin, HCT=Hematocrit, MCV=Mean corpuscular volume, MCH=Mean corpuscular hemoglobin, MCHC=Mean corpuscular hemoglobin concentration, RDW=Red cell distribution width, PLT=Platelets count, EBN=Edible bird’s nest

In contrast, EBN had no significant effects on other hematological indices, such as RBC count, HGB, HCT, MCH, and MCHC. In addition to the significant effects of EBN supplementation on neutrophil counts (5.0% increase relative to Premier E^®^ and control groups), reflecting a better immunomodulating response, other leucocyte counts were not significantly altered.

## DISCUSSION

Given the promising effects of EBN on hepatorenal metabolism as reported in previous studies by Yusop *et al*. [13] and Tan *et al*. [16], this study evaluated the safety of EBN supplementation in ameliorating the changes occurring in the cardiorespiratory, neuromuscular, and endocrine systems of Arabian (Athletic) race stallions’ during exercise, as well as the anti-inflammatory and immunomodulatory effects.

In the first experiment, the results of the 12-day trial showed that oral administration of EBN to healthy horses was not associated with any significant adverse effects. This was based on comparisons of vital parameters, hematological indices, electrolytes, liver, and kidney function between the experimental groups and with respect to time. The main reason for performing the first experiment was to validate the safety of EBN supplementation in healthy horses before investigating its anti-inflammatory and immunomodulating effects in exercising horses.

For instance, safety issues such as high nitrate content [17], EBN adulteration [18], and traces of heavy metals and pathological organisms [19] have been reported following the use of EBN. Tan *et al*. [16] reported that EBN Malaysia is characterized by a safe nutritional profile that is free of heavy metals and has acceptable nitrite and nitrate levels. Although the profile of EBN used in this study was not evaluated, our results show its safety as a supplement in healthy horses. All vital and hematological parameters remained within normal ranges for healthy horses throughout the study. Although the PLT and iron (Fe) levels were significantly higher at weeks 1 and 2 in the horses supplemented with EBN compared to the control group, the values were within the normal limits. A recent study demonstrated significant changes in thrombocytes after racing [19]. A possible explanation for the decreased count of thrombocytes in the control group is blood loss. Significant gastrointestinal blood loss has been reported in marathon runners [20]. Similar effects may be triggered in horses after an endurance race, as reflected by the decreases in PLT, Fe, RBC, and plasma proteins. However, the latter parameters were not statistically significant. Oral supplementation with EBN in athletic horses may ameliorate this exercise-induced gastrointestinal blood loss, but the underlying mechanism remains unknown.

In terms of electrolyte content, the sodium ion levels in the EBN-supplemented group were significantly lower than those in the control group at week 3. A significant decrease in sodium and chloride levels may also occur during exercise [20, 21]. Horses secrete hypertonic sweat and lose sodium, potassium, and chloride through perspiration [22]. Hyponatremia arising from either an endurance race or intense exercise is more likely a consequence of a large loss of water and sodium through sweating, which is usually associated with reduced water intake.

Organ function tests were also performed to explore the potential benefits of EBN supplementation on athletic horse performance and general health status. The control group exhibited signs of muscular damage based on elevated CK and AST levels and alterations in the hepatic system, with greater total bilirubin levels than the EBN group. These alterations reflect muscular injury, which is expected because the horses in this study were athletic. The lower levels of CK, AST, and total bilirubin in the EBN-supplemented group indicate the hepatorenal protective effects of EBN, as reported in previous studies by Albishtue [23] and Quddus *et al*. [24]. This is considered a key finding given the importance of assessing muscle-damage enzymes for monitoring possible injuries during the training of horses [12]. Another vital factor to consider in monitoring racehorses’ serum biochemistry is overtraining syndrome, which may trigger an increase in muscle enzymes (AST and CK) [23]. Thus, EBN supplementation may help address this overtraining syndrome because it is a common problem in horses undergoing strenuous training, as observed in the present study of Arabic stallion horses.

Overall, the reno-protective effect of EBN has been linked to the presence of SA and epidermal growth factors [25, 26], which are involved in repairing damaged tissues, maintaining renal tissue integrity, and downregulating the pro-inflammatory pathway. To elucidate the role of these mechanisms and their contribution to the positive effects of EBN supplementation, the second experiment in this study evaluated the levels of SA in various groups of athletic Arabian horses exposed to intense exercise.

In the second experiment, SA levels did not differ between the treatment and control groups at weeks 2 and 3 post-exercise. However, the horses supplemented with EBN had significantly higher SA levels at week 4 post-exercise compared to the groups fed Premier E^®^ and control. SA, an acetylated derivative of neuraminic acid, is present in animal tissues and body fluids and is an important biomarker in inflammatory conditions [27, 28]. A recent study conducted among horses infected with piroplasmosis found increased serum SA concentrations in affected groups compared with control animals [28], linked to changes in receptor-ligand interactions, a vital mechanism during the inflammatory response [29]. Moreover, EBN contains higher bioactive compounds, such as SA, which help improve metabolism, immune function, and physiological functions [11, 30]. Thus, the increase in SA levels in horses supplemented with EBN may indicate the ability of EBN to elicit an inflammatory response following exhausting exercise. In addition, the maintenance of SA levels at early periods of the experiment (weeks 2 and 3) might indicate the dose-dependent effect of EBN, consistent with reports from a previous study by Quddus *et al*. [24] that evaluated the effects of EBN on the regulation of antioxidant genes.

We also evaluated the hematological parameters of each treatment group at the end of the exercise test. On completing the exercise test at the end of the 4^th^ week, no significant differences in the RBC, HGB, HCT, MCH, and MCHC were observed among the EBN, Premier E^®^, and control groups. The effects of exercise on erythrocyte indices may vary depending on the work intensity, environmental conditions, fitness and training levels, and breed of the horse [31]. For instance, Krumrych [32] found significant changes in RBS, HGB, and HCT in different breeds of horses after an exercise test. In contrast, other researchers reported changes in hematological profiles in yearling trotters undergoing training programs [8, 31]. In the present study, the fact that these RBC indices were within normal values signified that both EBN and Premier E^®^ supplements were able to facilitate proper blood cell function and maintain homeostasis after exercise. Ko *et al*. [31] also reported that RBCs in riding ponies undergo mechanisms similar to those that attenuate oxidative stress after exercise, specifically oxidative stress-dependent impairment of RBC stability. Although we only evaluated the post-exercise hematological indices, the upward regulation of the total SA concentration in horses supplemented with EBN indicated its potential for alleviating oxidative-related effects.

EBN supplementation positively affected PLTs and RDW, which are indicative of thrombocyte stability in athletic horses. Changes in erythrocyte indices such as RBC count and RDW at post-exercise periods have been reported in horses [15, 31], but the underlying mechanisms are not well understood. RDW is an index measure of the range of variation of erythrocyte volume, which can also be calculated from the MCV. A phenomenon known as anisocytosis – an increase in the percentage of circulating immature erythrocytes that may result from oxidative stress-related changes affecting the survival of RBCs [32–34]. Overall, the significant increase in the few RBC indices observed in the EBN and Premier E^®^ supplemented groups might be related to the mobilization of splenic erythrocytes, leading to increased oxygen transport capacity. However, the mechanism underlying the changes in RBC indices in exercise horses supplemented with EBN is poorly understood, but the attenuation of oxidative-related changes could be a contributing factor. Further studies are required to understand how EBN supplementation influences oxidative changes that are important for the formation and homeostasis of RBC indices.

Furthermore, EBN supplementation resulted in a slightly higher neutrophil count post-exercise. Horses subjected to intensive and exhausting training undergo significant post-exercise leukocytosis characterized by neutrophilia [32] and lymphopenia [35]. In this study, the WBC indices were measured only after exercise; hence, comparing these results with other studies requires caution. Since the neutrophil counts post-exercise were within the normal range in horses supplemented with EBN, the result does not suggest any pathological changes. Nevertheless, EBN can stimulate the inflammatory response, and changes in WBCs are accompanied by impairment of immune cell function manifested by a temporary decline in the phagocytic and bactericidal activity of polymorphonuclear cells [32]. Results regarding the other WBC indices may be influenced by the type of exercise test to which the horses were exposed. Whereas horses in previous studies went through 80-km endurance exercise, the horses in this study conducted 10 min of canter at weekly intervals [32, 33]. Moreover, complex mechanisms underlie the diverse effects of physical exercise on immune function with the participation of cytokines, catecholamines, cortisol, and growth hormones [28].

## CONCLUSION

This study demonstrates the safety and efficacy of EBN supplementation in Arabian race stallions. The findings revealed that EBN supplementation significantly reduced exercise-induced hepatic and muscle stress markers, including creatine kinase, total bilirubin, and AST, while enhancing sialic acid levels, indicating improved anti-inflammatory responses. Hematological indices such as platelet count, MCV, and RDW showed favorable changes, reflecting potential immunomodulatory benefits. Importantly, no adverse effects on vital parameters or metabolic markers were observed, highlighting the safety of EBN supplementation.

The study employs a rigorous experimental design, including both safety and efficacy evaluations, providing a comprehensive assessment of EBN supplementation. Multiple physiological and biochemical parameters were analyzed, offering robust evidence of EBN’s protective and restorative effects. The focus on a well-defined population of Arabian race stallions contributes to the specificity and relevance of the findings for athletic horses.

However, the small sample size and short supplementation period limit the generalizability of the results to other equine populations or longer-term effects. The absence of molecular analyses prevents a deeper understanding of the mechanisms underlying EBN’s anti-inflammatory and immunomodulatory effects. In addition, the study focused exclusively on Arabian race stallions, which may not represent other horse breeds or exercise regimens.

Future studies should involve larger-scale research with diverse equine populations and extended supplementation periods to validate and expand the findings. Investigating the molecular mechanisms of EBN’s effects on oxidative stress, inflammation, and immune modulation using advanced biochemical and genetic tools would provide deeper insights. Long-term impacts of EBN supplementation on athletic performance, recovery, and health outcomes in horses subjected to various training and environmental conditions should also be explored. Assessing optimal dosages and formulations of EBN for maximal efficacy in different applications, such as performance enhancement and recovery support, would further enhance its utility. This study provides a foundation for incorporating EBN as a functional dietary supplement in equine sports management, promoting health and performance in athletic horses.

## AUTHORS’ CONTRIBUTIONS

KOA and KHA: Field and laboratory work, data analysis, curation, interpretation, and drafted the manuscript. ASB and SJL: EBN samples production, advised on sialic acid plasma concentrations method, and reviewed and revised the manuscript. MBS: Data analysis, curation, interpretation, and reviewed and revised the manuscript. NY: Study design, supervision, and administration. All authors have read and approved the final manuscript.
